# Near-Complete Genome Sequences of Vesicular Stomatitis Virus Isolates from the 2020 Outbreak in Kansas

**DOI:** 10.1128/MRA.01454-20

**Published:** 2021-02-18

**Authors:** Tyler Doerksen, Edward Bird, Jamie Henningson, Rachel Palinski

**Affiliations:** a Kansas State Veterinary Diagnostic Laboratory, Kansas State University, Manhattan, Kansas, USA; Portland State University

## Abstract

Here, we report the near-complete genome sequences of vesicular stomatitis virus (VSV) serotype Indiana isolates from the 2020 U.S. outbreak. The sequences were obtained from swabs collected from Kansas horses in July and August. The four genome sequences help improve our understanding of VSV outbreak dynamics in the United States.

## ANNOUNCEMENT

Vesicular stomatitis virus (VSV; genus *Vesiculovirus*, family *Rhabdoviridae*) is a negative-sense, single-stranded RNA virus that commonly causes vesicular disease outbreaks in cattle, sheep, goats, pigs, and horses in the Western Hemisphere. Vesicular stomatitis (VS), caused by VSV, manifests with vesicles around the mouth, tongue, ears, and coronary band (1, 2). Since the clinical presentation is indistinguishable from that of other high-consequence vesicular diseases, such as foot-and-mouth disease, rapid detection and diagnosis are essential. The two major VSV serotypes, Indiana (VSIV) and New Jersey (VSNJV), cause cyclical epidemics in the United States thought to originate from regions of Central America where VSV is endemic (3–5). The most recent U.S. VSV outbreak started in 2019 and affected 7 states (Colorado, Nebraska, New Mexico, Oklahoma, Texas, Utah, and Wyoming) that year, expanding unusually far east the subsequent year (2020), affecting 8 states in total (Arizona, Arkansas, Kansas, Missouri, Nebraska, New Mexico, Oklahoma, and Texas) (6).

Following the 2020 outbreak of VS in Kansas, lesion swabs were collected from horses with clinical signs of vesicular disease throughout southeastern Kansas and used in this study.

At Kansas State Veterinary Diagnostic Laboratory (KSVDL), RNA was extracted using the QiaAmp viral RNA minikit (Qiagen), followed by first-strand cDNA synthesis with random hexamers (Superscript III; Invitrogen) and second-strand synthesis with a nondirectional polymerase (NEBNext Ultra nondirectional RNA second-strand synthesis module) (7). Primers were removed (HighPrep PCR cleanup system; MagBio), and libraries were prepared using the Nextera XT dual-indexing kit. Libraries were run on the Illumina MiSeq platform using a 300-cycle v2 cartridge. All sample preparation steps were performed following the manufacturer’s protocols.

Raw reads were trimmed, *de novo* assembled into contigs, and analyzed with BLASTn. The closest reference was identified as VSIV isolate IN0919COB (GenBank number MT437285), collected in 2019 from a bovine in Colorado (8). Trimmed reads were aligned to IN0919COB, and a consensus sequence was extracted. All bioinformatics steps were performed using default parameters in CLC Workbench v 20.0.3. The genomes were designated IN0720KSE, IN0720KSE2, IN0720KSE3, and IN0820KSE. Sequencing metrics are specified in Table 1.

**TABLE 1:** Sequencing metrics

Parameter	Data for strain:			
	IN0720KSE	IN0720KSE2	IN0720KSE3	IN0820KSE
No. of reads	418,744	2,135,296	5,873,128	1,541,424
Avg read length (bp)	136	136	140	139
No. of contigs	444	536	1,948	767
Avg coverage (×)	30.6	179	100	178
Genome length (bp)	11,051	11,162	11,134	11,164
% similarity to reference	99.81	99.75	99.79	99.76

We report the first VSV genome sequences from the 2020 U.S. outbreak season. The four genomes have a GC content of 41.8% and five open reading frames (N, NS, M, G, and L) encoding proteins of the expected sizes (423, 266, 230, 512, and 2,110 amino acids) (9, 10). The genomes are 99.90% to 99.99% identical to each other and 99.75% to 99.81% identical to the IN0919COB reference, correlating to 1- to 11-nucleotide (nt) and 22- to 28-nt differences, respectively. Nucleotide differences between the samples are present in N (*n* = 2), G (2), L (6), and noncoding regions (1). Compared with the reference, nucleotide differences occur in N (2), NS (1), M (1), G (0), L (13), and noncoding regions (10).

Advances in deep sequencing technologies have allowed rapid sequencing of whole genomes concurrent with outbreaks. These advances present unique opportunities to analyze and predict outbreak dynamics in real time, thereby governing a subsequent response. The four near-complete genome sequences discussed in this study will fill key knowledge gaps associated with the molecular evolution of the most recent (2020) VSV outbreak.

### Data availability.

The consensus genome sequences were deposited in GenBank under accession numbers MW373776, MW373777, MW373778, and MW373779. The deep sequence reads were deposited in NCBI Sequence Read Archive (SRA) under project number PRJNA685396. This project references the first version of the sequences.
